# Nucleoside reverse transcriptase inhibitor-induced rat oocyte dysfunction and low fertility mediated by autophagy

**DOI:** 10.18632/oncotarget.23243

**Published:** 2017-12-13

**Authors:** Li Tang, Shengfu Yang, Huawei Wang, Hai Gu, Xueshan Xia, Yue Feng, Zexing Yang, Shuhua Zhao, Cunmei Su, Zhenfang Su, Kunhua Wang

**Affiliations:** ^1^ Department of Reproduction and Genetics, The First Affiliated Hospital of Kunming Medical University, Kunming, 650032, Yunnan Province, China; ^2^ Department of Reproductive Medicine, The First People’s Hospital of Yunnan Province, Kunming, 650032, Yunnan Province, China; ^3^ Department of Pharmacy, The First People’s Hospital of Yunnan Province, Kunming, 650032, Yunnan Province, China; ^4^ Yunnan Institute of Digestive Disease, The First Affiliated Hospital of Kunming Medical University, Kunming, 650032, Yunnan Province, China; ^5^ Department of Life Science, Kunming University of Science and Technology, Kunming, 650093, Yunnan Province, China

**Keywords:** low fertility, NRTIs, mitochondrial toxicity, autophagy

## Abstract

Low fertility is one of the most common side effects caused by nucleoside reverse transcriptase inhibitors (NRTIs), whereas the molecular mechanism underlying this process were largely unclear. This study was conducted to investigate whether autophagy plays a role in NRTIs-induced oocyte dysfunction and low fertility in female rat. Both *in vivo* and *in vitro* experiments were conducted. For the *in vivo* experiment, female adult Sprague-Dawley rats were subjected to zidovudine (AZT) and lamivudine (3TC) intragastric treatment for 3, 6, 9, and 12 weeks; a control was also set. Oocytes were collected for maturation evaluation, *in vitro* fertilization and mitochondrial function assays, and apoptosis and autophagy analysis. For the *in vitro* experiment, oocytes were collected and assigned to the control, 3-methyladenine (3-MA, an effective autophagy inhibitor), AZT, AZT+3-MA, 3TC, and 3TC+3-MA groups. The oocytes were cultured with the abovementioned drugs for 24, 48, and 72 h and then, subjected to the same assays as in the *in vivo* study. The results showed a significant time-dependent decrease in oocyte maturation-related maker levels, oocyte cleavage rate, blastocyst formation rate, mitochondrial DNA copy number and adenosine triphosphate level, and apoptosis, and a significant increase in the reactive oxygen species levels (all *P*-values < 0.05), in both the *in vivo* and the *in vitro* experiments. These changes, except for the changes in the oocyte maturation-related markers, were partially attenuated by 3-MA. In conclusion, we demonstrated that NRTIs can cause rat oocyte dysfunction and low fertility, and this damage was, at least partially, mediated by autophagy.

## INTRODUCTION

Acquired immunodeficiency syndrome (AIDS) is a serious infectious disease that has affected human health across the world [1]. Nucleoside reverse transcriptase inhibitors (NRTIs) are the first class of antiretroviral drugs in clinical use that by effectively inhibiting the conversion of the virus significantly prolong the lifespan of AIDS patients [2]. However, a large number of side effects of NRTIs, such as metabolic disorders, hepatic damage, kidney injury, and low fertility, have been observed [3]. Mitochondria are responsible for producing energy for cells; thus, NRTI-associated mitochondrial DNA (mtDNA) toxicity and mutations, as one of the side effects of NRTIs, could induce the dysfunction of mitochondria and worsen the condition of AIDS patients [4]. The reduction of the number of copies of mtDNA is a widely known effect of mitochondrial toxicity with NRTIs [5]. Previous works have revealed that the NRTI stavudine can reduce the mtDNA copy number and invoke mtDNA toxicity in mice oocytes [6]. Further, a reduction of mtDNA copy number was detected in the peripheral blood [7] and was associated with the low fertility of AIDS patients [8].

The DNA polymerase γ hypothesis has been widely used to account for the reduction of mtDNA copy number and the mitochondrial toxicity for AIDS patients treated with NRTIs [9]. An imbalance of oxidative stress and the dysfunction of histone and the DNA repair system have been found to be associated with increased mtDNA mutation and to aggravate the disease progression of AIDS patients [9]. Mitochondrial toxicity can decrease oocyte reserve [10] and reduce the spontaneous fertilization rate, *in vitro* fertilization rate, and pregnancy rate following embryo transfer [11, 12]. Moreover, research focused on NRTI-induced cell apoptosis has revealed that apoptosis may be a factor in the process of NRTI-induced mitochondrial toxicity [12, 13].

However, the mechanism underlying the low fertility of AIDS patients treated with NRTIs is still unclear. Considering the lack of HIV receptors in an oocyte and its surrounding granulosa cells [8], we have hypothesized that the reduction of the mtDNA copy number in the oocytes of an AIDS patient treated with highly active antiretroviral therapy (HAART) may be induced by NRTIs directly and not by the HIV infection. Reactive oxygen species (ROS) are mainly generated in cells during the process of oxidative phosphorylation, which causes depolarization damage to mitochondria [14]. Autophagy plays a crucial role in eliminating the damaged mitochondria and programmed cell apoptosis [15, 16], to maintain the stability of the intracellular environment [17–19]. Thus, we speculate that the reduction of the mtDNA copy number and the dysfunction of oocytes in AIDS patients treated with NRTIs may be mediated by autophagy. Oocyte development is a complicated process which is susceptible to multiple internal and external factors. Maturation is the last stage of oocyte development and only those matured oocytes have the capacity of fertilization [20, 21]. However, until now, no study has evaluated the effect of NRTIs on oocyte maturation.

In this study, we aimed to examine the effect of two commonly used NRTIs, zidovudine (AZT) and lamivudine (3TC) on rat oocyte function from the perspectives of *in vitro* fertilization, oocyte maturation, mtDNA copy number, ROS level, and apoptosis, and to investigate whether AZT- and 3TC-induced oocyte dysfunction was mediated by the autophagy pathway.

## RESULTS

### Effect of NRTIs on oocyte maturation

**Figure 1:**
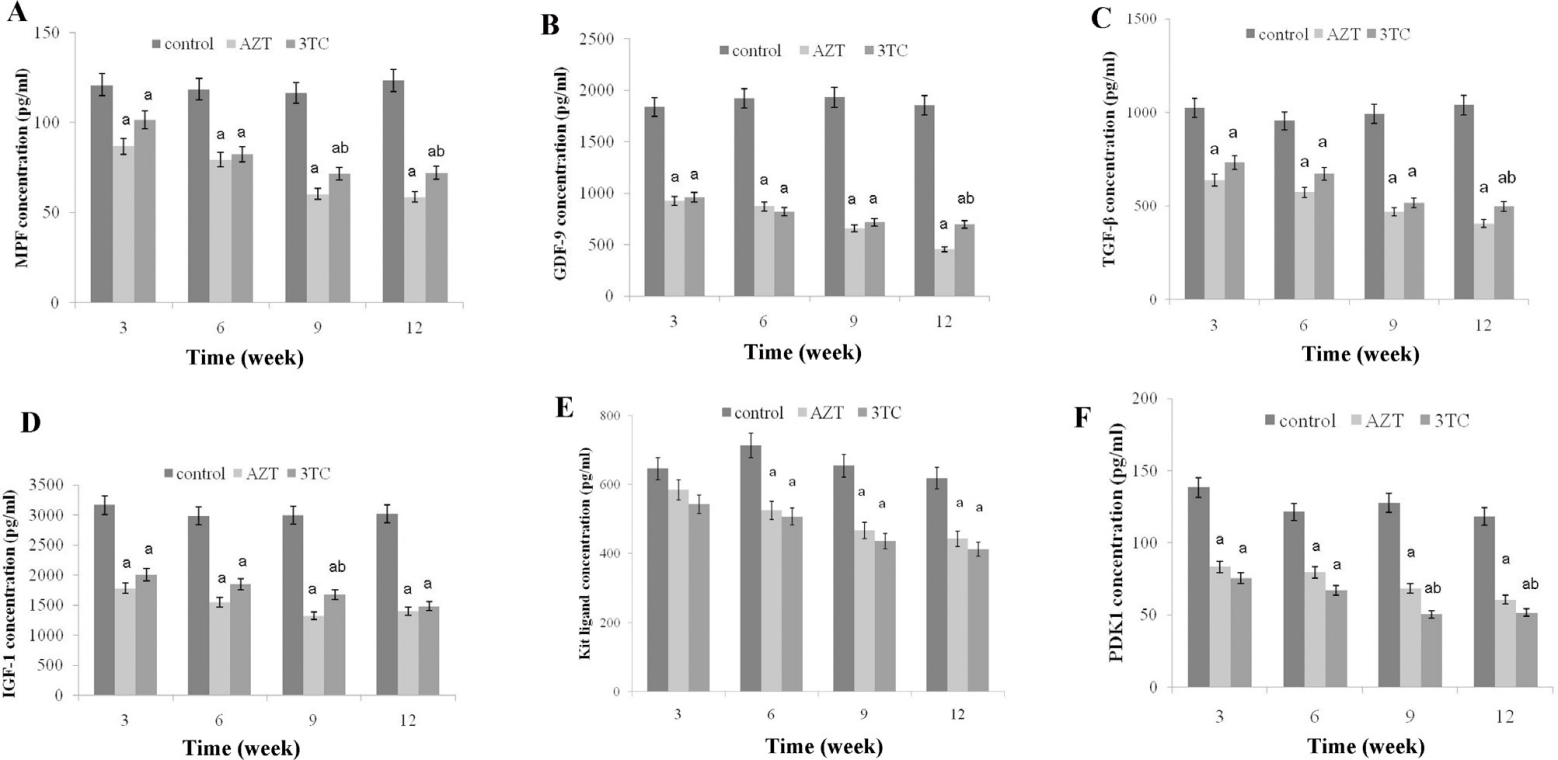
Maturation of rat oocytes was evaluated by measuring the levels of MPF, GDF-9, TFG-β, IGF-1, KL, and PDK1 (**A–F**) with enzyme-linked immunosorbent assay (ELISA), for the *in vivo* experiment. The rats were treated with AZT and 3TC for 3, 6, 9, and 12 weeks, and then, their oocytes were isolated for measuring the aforementioned biomarkers. Here, a represents the statistically significant differences with the control group (*P* < 0.05), and b denotes the statistically significant differences between the AZT and the 3TC groups (*P* < 0.05).

**Figure 2:**
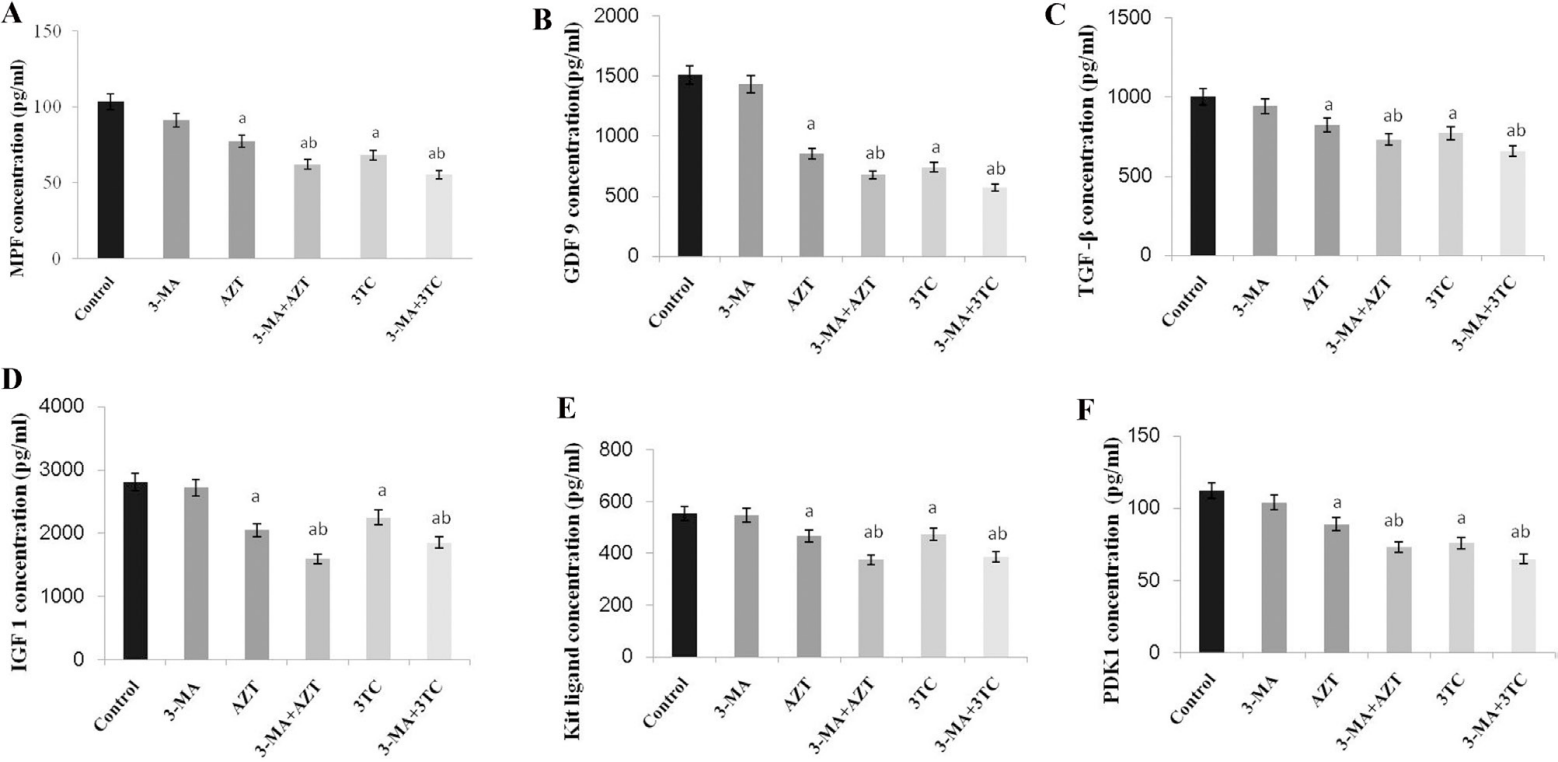
Maturation of rat oocytes was evaluated by measuring the levels of MPF, GDF-9, TFG-β, IGF-1, KL, and PDK1 (**A–F**) with ELISA, for the *in vitro* experiment. The oocytes were treated with AZT and 3TC for 24, 48, and 72 h. Here, a represents the statistically significant differences with the control groups (*P* < 0.05), and b denotes the statistically significant differences between the AZT and the AZT+3-MA groups, and between the 3TC and the 3TC+3-MA groups (*P* < 0.05).

MPF, GDF-9, TGF-β, IGF-1, Kit ligand, and PDK1 were measured to evaluate the maturation of rat oocytes. The results of the *in vivo* study showed that all the oocyte maturation-related markers decreased significantly after the AZT and 3TC treatment when compared with the control at all time points (all *P*-values < 0.05) (Figure 1). Note that the levels of these markers at week 9 were similar to those at week 12. Our data indicated that AZT and 3TC, two major NRTIs, could suppress the maturation of rat oocytes *in vivo*, and the effect was time-dependent. For the *in vitro* study, oocytes were cultured with AZT, 3TC, and/or 3-MA for 24 h and then tested. The results revealed that the levels of all the abovementioned markers decreased significantly in the AZT, 3TC, AZT+3-MA, and 3TC+3-MA groups (all *P*-values < 0.05) in comparison with the control. However, 3-MA was found to aggravate the effect of NRTIs on oocytes, as shown in Figure 2.

### NRTIs reduced rat fertility

**Figure 3:**
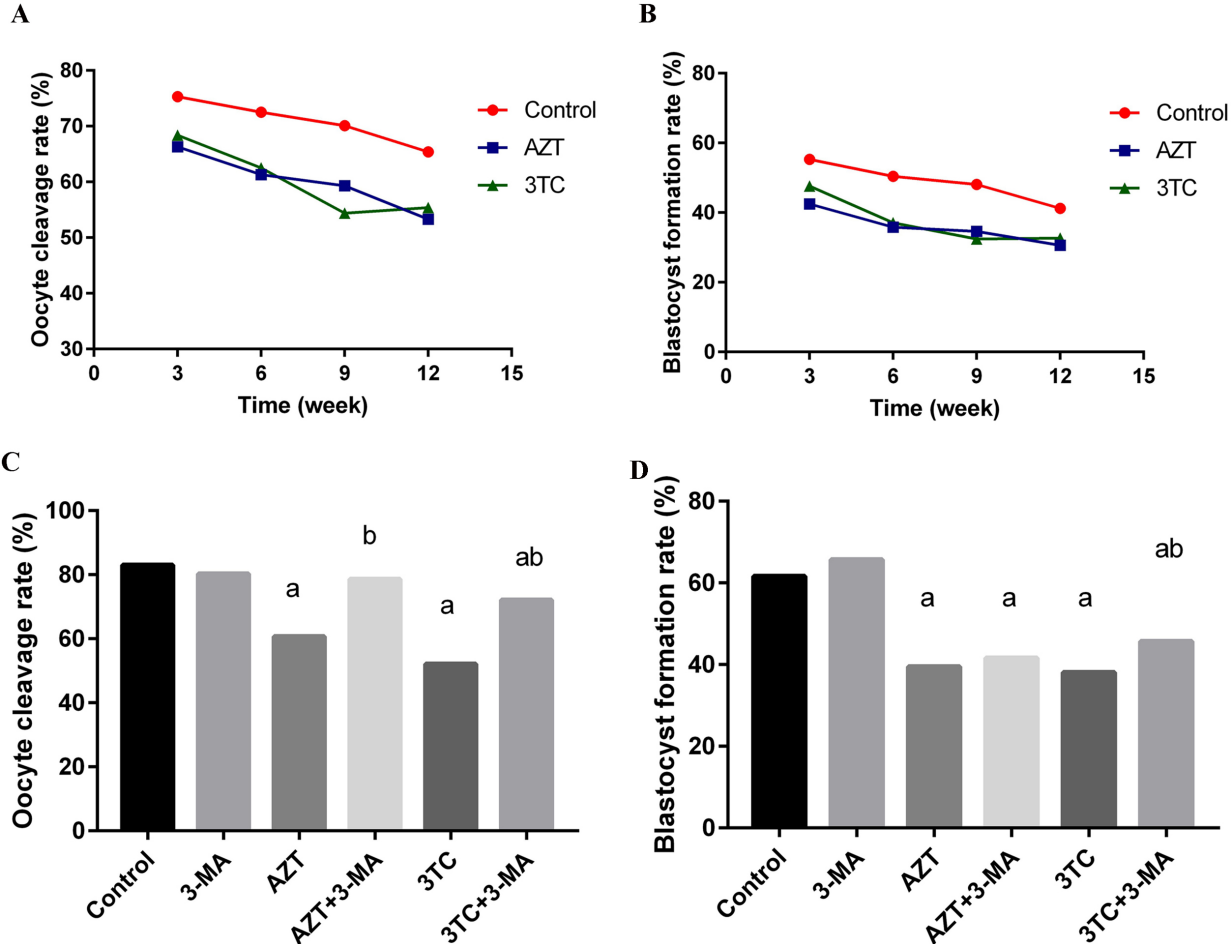
Oocyte cleavage rate and blastocyst formation rate in different treatment groups, for the *in vivo* experiment (**A** and **B**) and the *in vitro* experiment (**C** and **D**). The rates in the AZT and 3TC groups were all significantly lower than those in the controls, at different time points, for the *in vivo* experiment (all *P*-values < 0.05). Here, a represents the statistically significant differences with the control group (*P* < 0.05), and b denotes the statistically significant differences between the AZT and the AZT+3-MA groups, and between the 3TC and the 3TC+3-MA groups (*P* < 0.05).

The oocyte cleavage rate and the blastocyst formation rate are the direct indexes of fertility *in vitro*. In the *in vivo* study, we found that the oocyte cleavage rate and the blastocyst formation rate of rat oocytes reduced gradually with an increase in the duration of NRTI treatment. The rates in the AZT and 3TC groups were all significantly lower than those in the controls, at different time points (all *P*-values < 0.05), as shown in Figure 3A and 3B. In the *in vitro* study, a co-culture with AZT and 3TC also significantly decreased the oocyte cleavage rate and the blastocyst formation rate (*P* < 0.05). Further, 3-MA attenuated the decreased rates caused by AZT and 3TC, even though the difference in the blastocyst formation rate between the AZT+3-MA and the AZT group was not statistically significant (*P* = 0.17) (Figure 3C and 3D).

### Effect of NRTIs on mtDNA copy number and mitochondrial function

**Figure 4:**
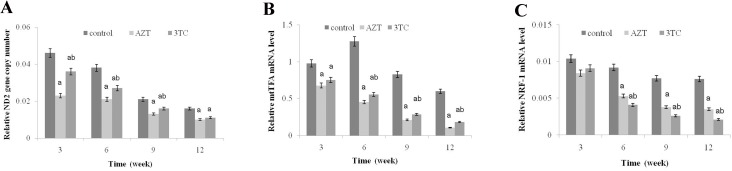
Effects of AZT and 3TC on the mtDNA copy number, and the mitochondrial function-related gene expression of mice oocytes, for the *in vivo* experiment The rats in the AZT and 3TC groups were suffering from AZT and 3TC for 3, 6, 9, and 12 weeks. The mtDNA copy number was evaluated by measuring the ND2 gene content (**A**). mtTFA (**B**) and NRF-1 (**C**) were measured to evaluate the mitochondrial function. Here, a represents the statistically significant differences with the control group at *P* < 0.05, and b denotes the statistically significant differences between the AZT and the 3TC groups at *P* < 0.05.

**Figure 5:**
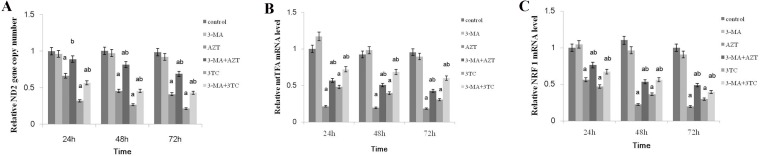
Effects of AZT and 3TC on the mtDNA copy number, and the mitochondrial function-related gene expression of mice oocytes, for the *in vitro* experiment The mtDNA copy number was evaluated by measuring the ND2 gene content (**A**). mtTFA (**B**) and NRF-1 (**C**) were measured to evaluate the mitochondrial function. Here, a represents the statistically significant differences with the control group (*P* < 0.05), and b denotes the statistically significant differences between the AZT and the AZT+3-MA groups, and between the 3TC and the 3TC+3-MA groups (*P* < 0.05).

**Figure 6:**
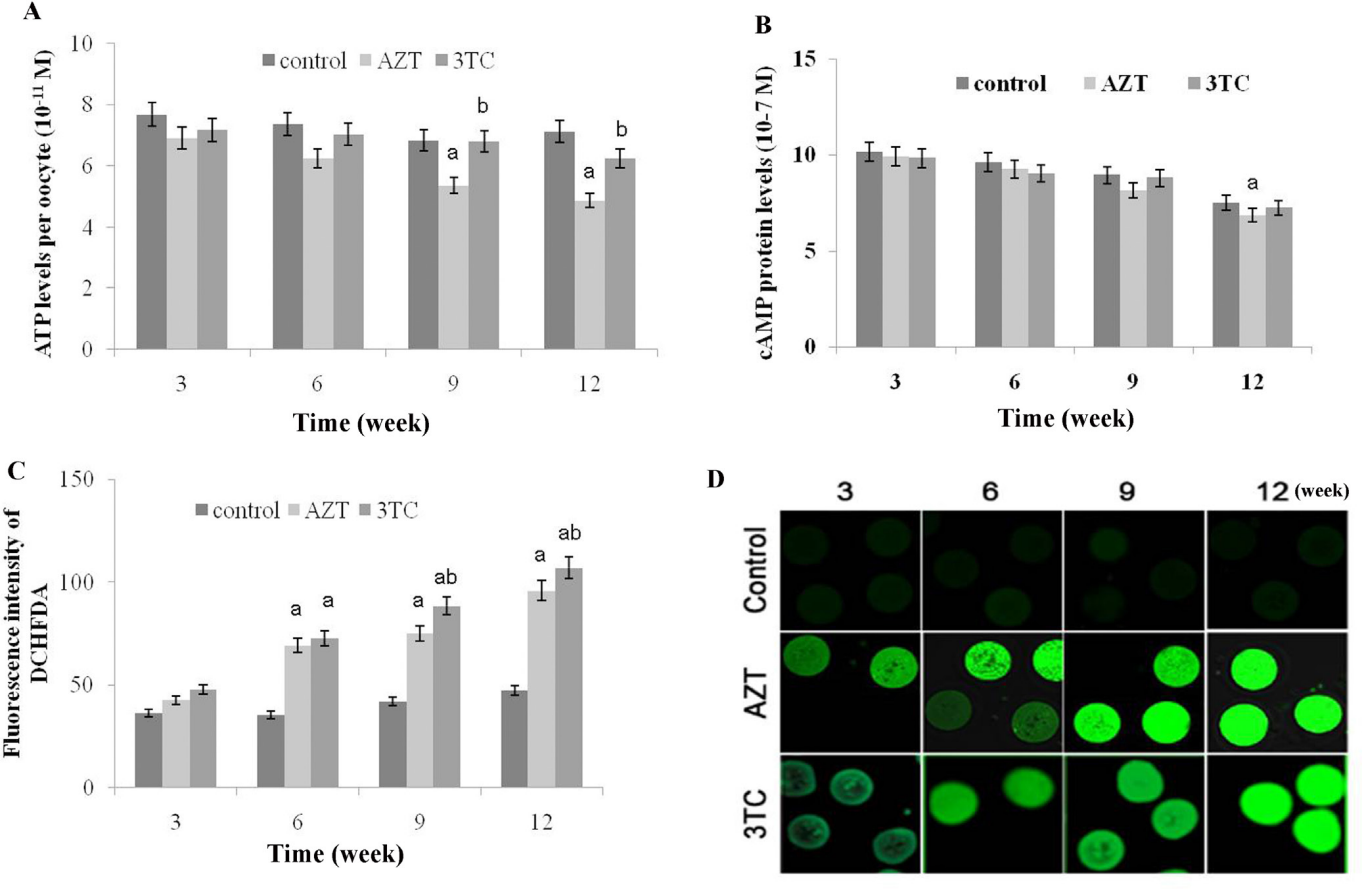
Effects of AZT and 3TC on the ATP, cAMP, and ROS levels of mice oocytes, for the *in vivo* experiment Rats were treated with AZT and 3TC for 3, 6, 9, and 12 weeks. The ATP level was measured with the ATP Assay KiT (**A**), and the level of cAMP was detected using luciferase (**B**). Further, the ROS level in the mitochondria was detected using dichloro-dihydro-fluorescein diacetate (**C**). The fluorescence intensity was in line with the ROS level (**D**). Here, a represents the statistically significant differences between the AZT, 3TC, and control groups at *P* < 0.05, and b denotes the statistically significant differences between the AZT and the 3TC groups at *P* < 0.05.

**Figure 7:**
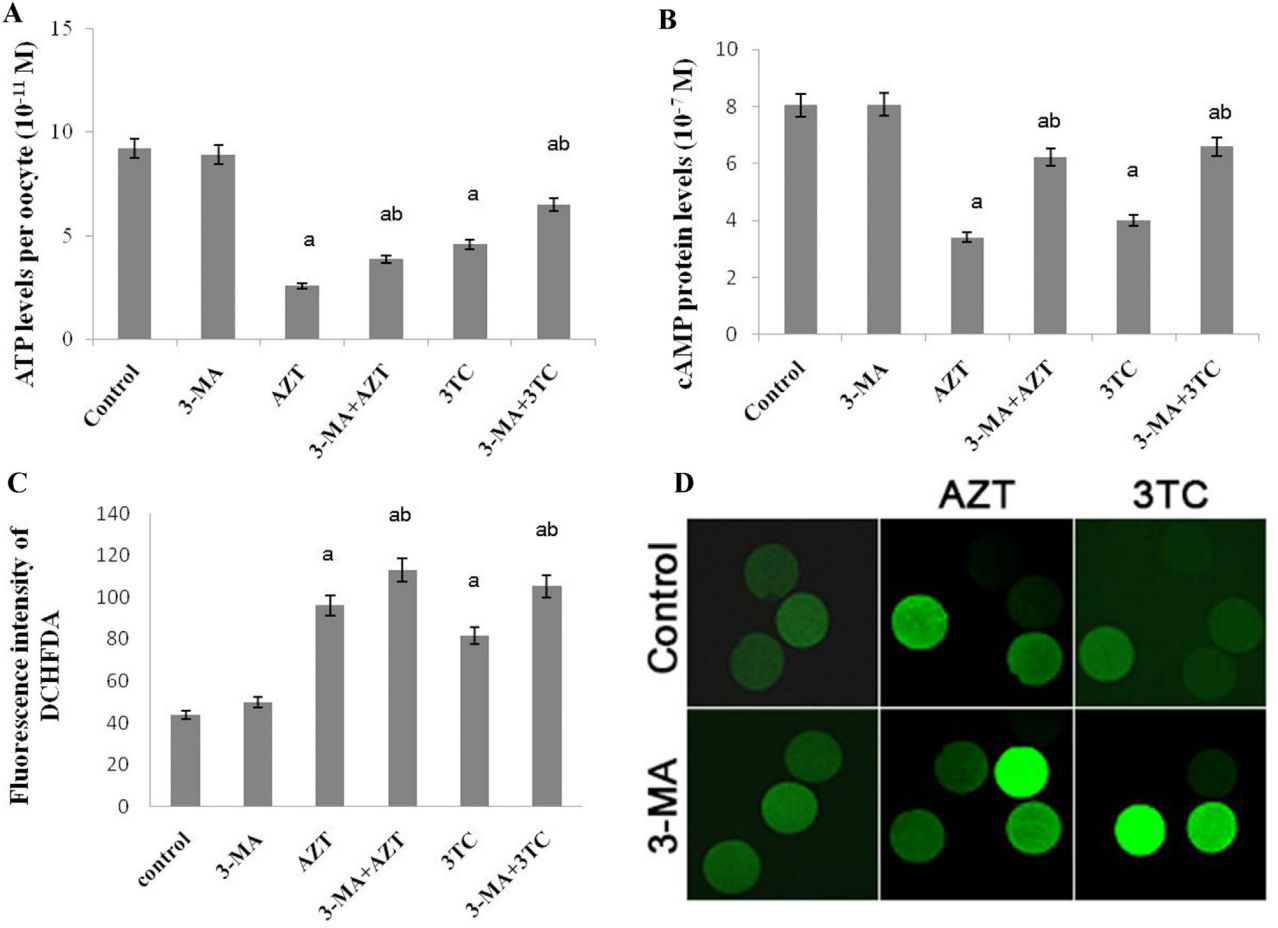
Effects of AZT and 3TC on the ATP, cAMP, and ROS levels of rat oocytes, for the *in vitro* experiment The ATP level was measured with the ATP Assay KiT (**A**), and the level of cAMP was detected with luciferase (**B**); the ROS level in the mitochondria was detected using dichloro-dihydro-fluorescein diacetate (**C**). The fluorescence intensity was in line with the ROS level (**D**). Here, a represents the statistically significant differences with the control group (*P* < 0.05), and b denotes the statistically significant differences between the AZT and the AZT+3-MA groups, and between the 3TC and the 3TC+3-MA groups (*P* < 0.05).

The conservative mitochondrial ND2 gene was analyzed to evaluate the mtDNA copy number. The results showed that the mtDNA copy number of rat oocytes treated with AZT and 3TC reduced drastically from 3 to 12 weeks, and the effect was considerably more severe in weeks 9–12 than in weeks 3–6. We also found that the mtDNA copy number in the AZT group was considerably lower than that in the 3TC group, in weeks 3–9 after treatment (*P* < 0.05 at each time point) (Figure 4A). The mtTFA and NRF-1 mRNA levels were tested to evaluate the mitochondrial function. The results showed that the expression levels of these two genes significantly downregulated after the AZT and 3TC treatment (all *P*-values < 0.05). Further, the downregulation was more obvious in weeks 9–12 than in weeks 3–6 after treatment in both groups. The downregulation in the AZT group was considerably more severe than in the 3TC group, as shown in Figure 4B and 4C. These results were confirmed by the *in vitro* study, which also showed decreased mtDNA copy number, mtTFA, and NRF-1 mRNA levels in the AZT and 3TC groups, in comparison with the control. Interestingly, all these effects were partially attenuated by 3-MA, irrespective of the AZT or 3TC treatment, as shown in Figure 5. Both the ATP and the cAMP levels of rat oocytes showed a decreasing trend with time in both treatment groups, whereas only the ATP level in the AZT group was statistically significantly lower than that in the control group at weeks 9 and 12 (both *P*-values < 0.05), and the cAMP level at week 12 was significantly decreased in the AZT group (*P* < 0.05) (Figure 6A and 6B). Moreover, the ROS levels were found to have increased gradually and statistically significantly in weeks 3–12 after the treatment (*P* < 0.05 at each time point), with a considerably higher level in the 3TC group than in the AZT group (Figure 6C and 6D). The *in vitro* study confirmed the results that AZT and 3TC obviously decreased oocyte ATP, cAMP levels, and increased ROS levels, whereas these alterations were not attenuated, but aggravated by 3-MA (all *P*-values < 0.05), as shown in Figure 7.

### NRTIs induced oocyte apoptosis

**Figure 8:**
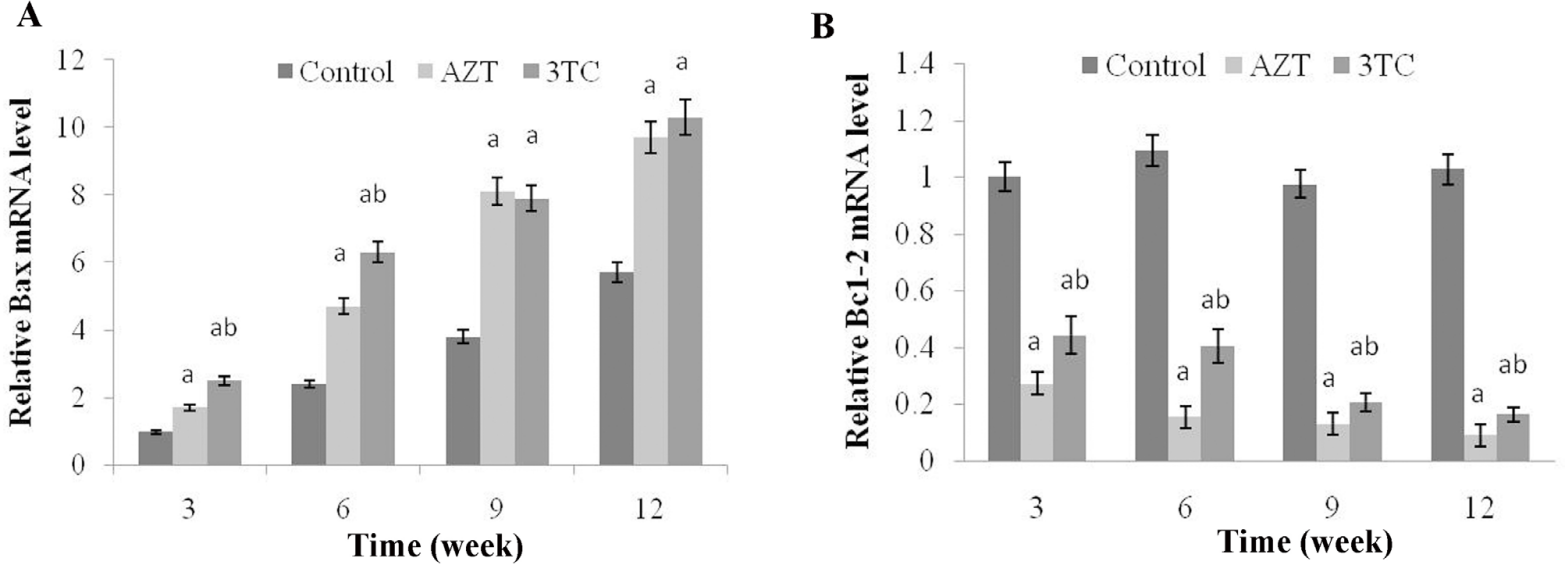
Effects of AZT and 3TC on the Bax and Bcl-2 mRNA expressions, for the *in vivo* experiment Here, (**A**) represents the statistically significant differences between the AZT, 3TC, and control groups at *P* < 0.05, and (B) denotes the significant differences between the AZT and the 3TC groups at *P* < 0.05.

**Figure 9:**
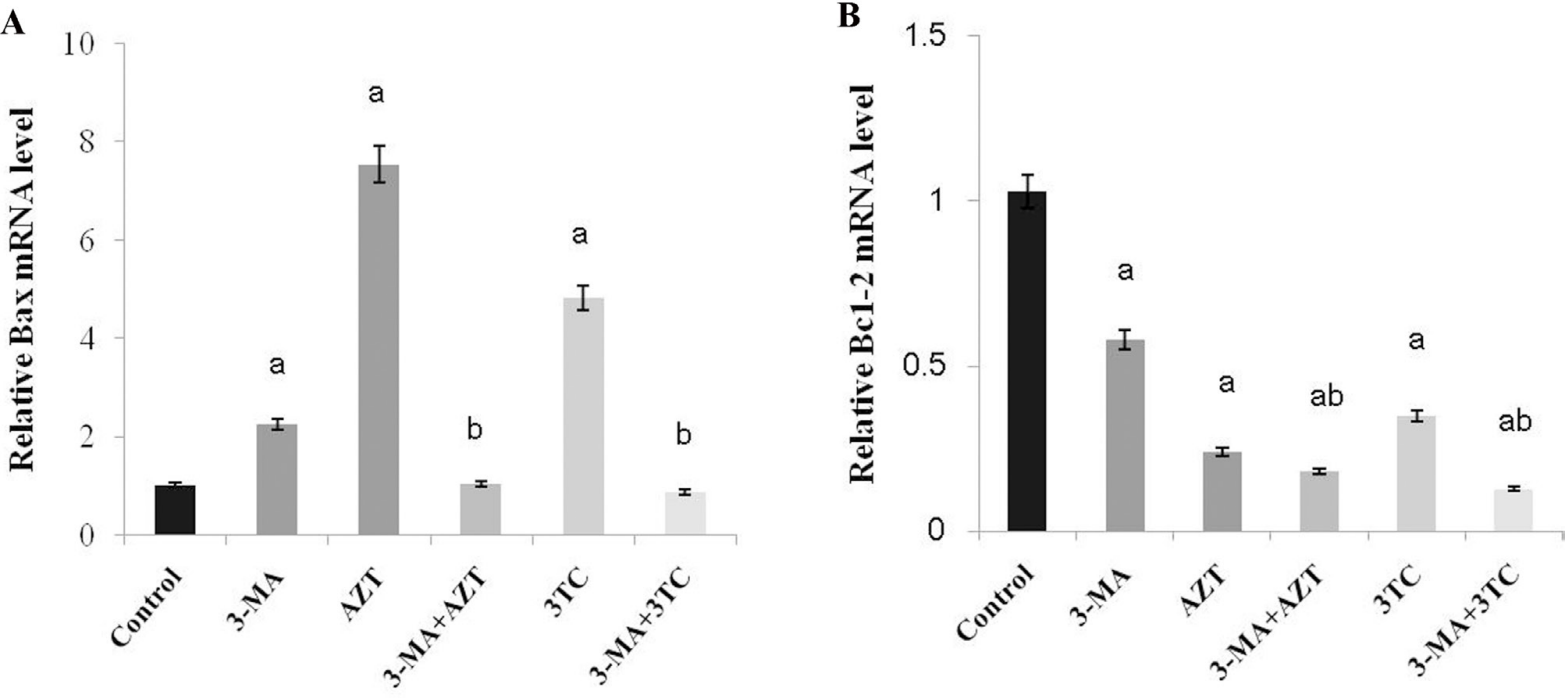
Effects of AZT and 3TC on the Bax and Bcl-2 mRNA expressions, for the *in vitro* experiment Here, (**A)** represents the statistically significant differences with the control group (*P* < 0.05), and (**B)** denotes the statistically significant differences between the AZT and the AZT+3-MA groups, and between the 3TC and the 3TC+3-MA groups (*P* < 0.05).

As shown in Figure 8, an obvious upregulation of Bax and downregulation of Bcl-2 mRNAs were found at weeks 3, 6, 9, and 12 after the NRTI treatment when compared with the control (*P* < 0.05 at each time point in both groups), in the *in vivo* study, indicating that NRTIs could induce oocyte apoptosis. These results were then confirmed by our *in vitro* study, which also showed increased Bax and decreased Bcl-2 mRNA levels in both the AZT and the 3TC treatment groups (both *P*-values < 0.05) (Figure 9). We also found that 3-MA could induce oocyte apoptosis and aggravate the effect of NRTIs on the oocytes.

### NRTIs induced autophagy in oocytes

**Figure 10:**
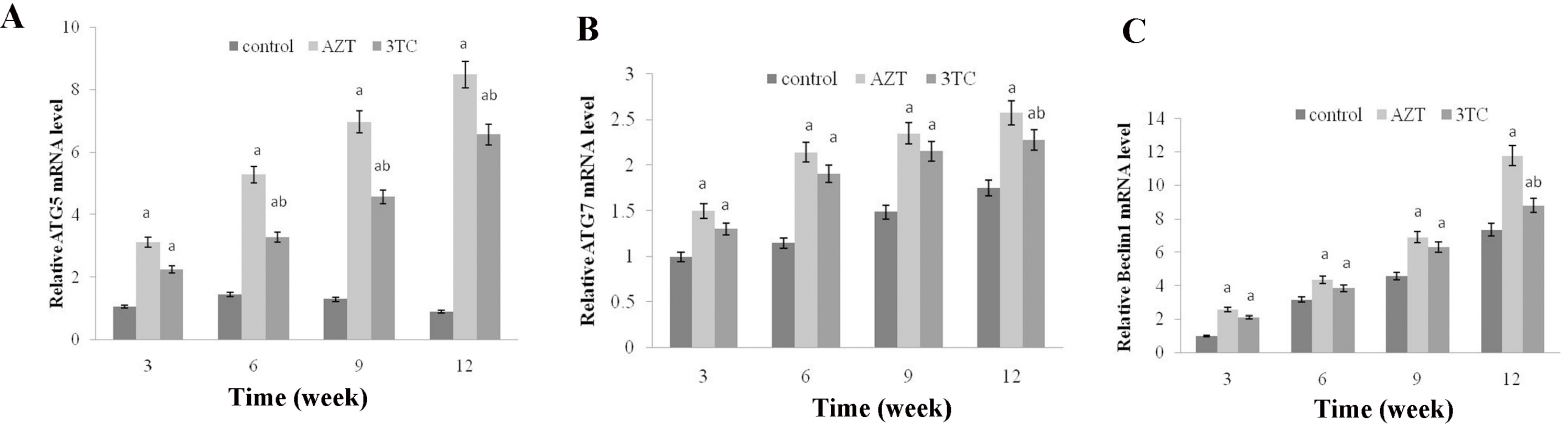
AZT- and 3TC-induced oocyte autophagy, for the *in vivo* experiment The ATG5 (**A**), ATG7 (**B**), and Beclin 1 (**C**) mRNA levels were assayed using qRT-PCR. Here, a represents the statistically significant differences between the AZT, 3TC, and control groups at *P* < 0.05, and b denotes the statistically significant differences between the AZT and the 3TC groups at *P* < 0.05.

**Figure 11:**
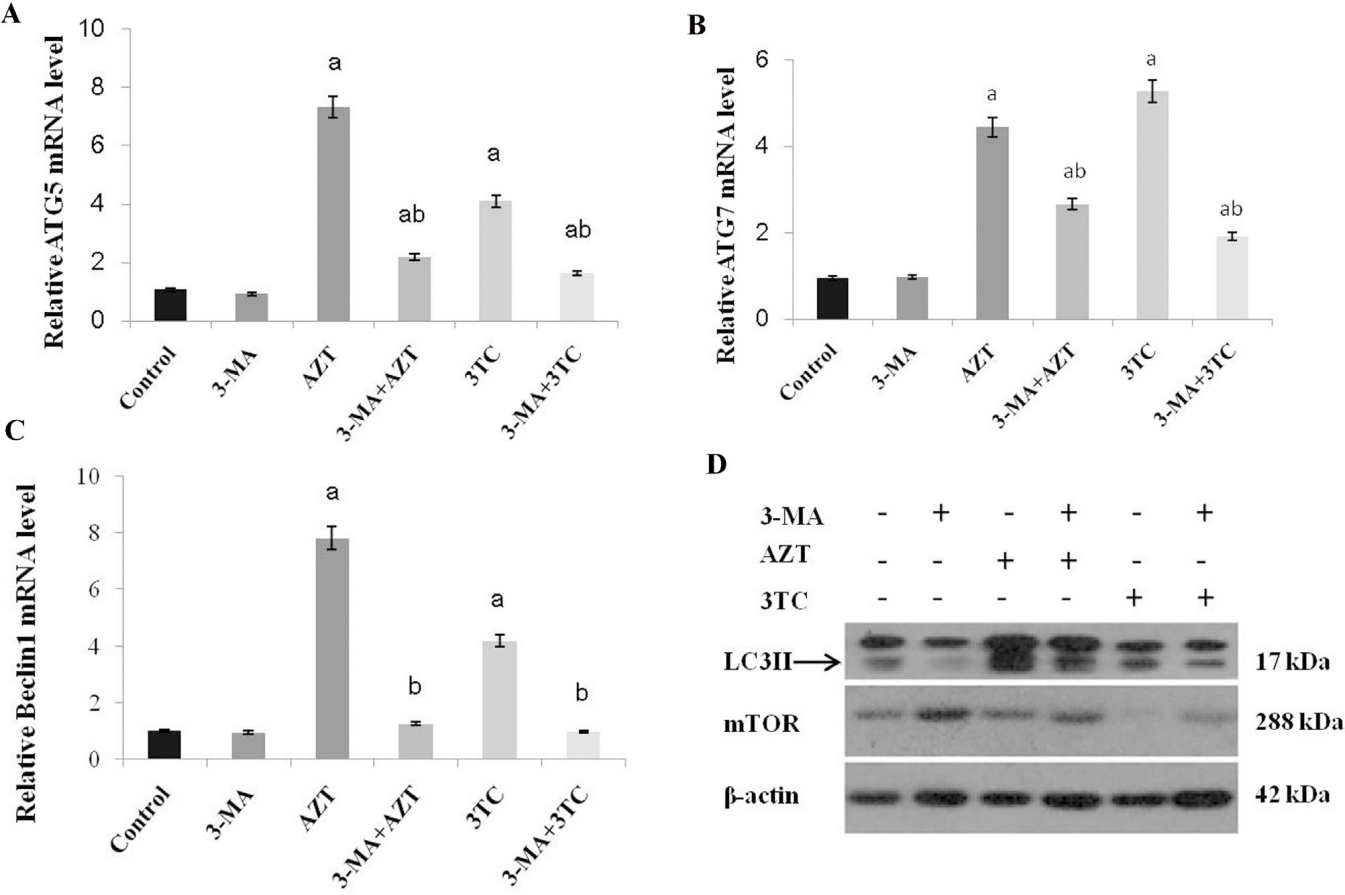
AZT- and 3TC-induced oocyte autophagy, for the *in vitro* experiment The ATG5 (A), ATG7 (B), and Beclin 1 (C) mRNA levels were assayed using qRT-PCR. LC3-II and mTOR were also analyzed in the different groups by using a Western blot (D). Here, a represents the statistically significant differences with the control group (*P* < 0.05), and b denotes the statistically significant differences between the AZT and the AZT+3-MA groups, and between the 3TC and the 3TC+3-MA groups (*P* < 0.05).

To evaluate whether autophagy was involved in the process of AZT- and 3TC-induced mitochondrial damage and decreased fertility, the expression levels of ATG 5, ATG 7, and Beclin1 mRNA were measured both *in vivo* (Figure 10) and *in vitro* (Figure 11). Both studies showed an upregulation of these mRNA levels in the AZT and 3TC groups (all *P*-values < 0.05 at each time point), and the values in the AZT group were considerably higher than those in the 3TC group. Note that the gene upregulation could be partially attenuated by 3-MA, irrespective of the AZT or 3TC treatment, as shown in the *in vitro* study (Figure 10). A further Western blot analysis showed that LC3-II in both the AZT and the 3TC groups was upregulated in the protein levels, when compared with the control. Moreover, the 3-MA treatment suppressed the upregulation of LC3-II in both the AZT and the 3TC groups. Contrasting results for the mTOR expression levels were also observed in both the AZT and the 3TC groups (Figure 11), indicating that autophagy mediated the oocyte damage caused by NRTIs.

## DISCUSSION

NRTIs are the first class of drugs for treating HIV infection and have shown considerable potential for inhibiting the HIV counts when incorporated with other drugs. However, NRTIs cause the low fertility of AIDS patients, including low sperm motility and sperm velocity in males [22], and a low oocyte retrieval rate in females [22]. Mitochondrial toxicity and mitochondrial DNA depletion have been suggested as one of the most critical causes for the low fertility of AIDS patients treated with NRTIs [8, 22]. Unfortunately, the detailed mechanism remains unclear. Previous work has revealed the increased oxidative stress condition and intracellular ROS levels in chronically HIV-1-infected patients [23]; these changes adversely affected the stability of the intracellular environment for the cells of AIDS patients and aggravated the disease progression [9]. In this study, we speculated that the reduction of the mtDNA copy number and the low fertility of oocytes treated with NRTIs may be medicated by autophagy. To test our hypothesis, the rat and rat oocytes were treated with AZT and 3TC *in vitro* and *in vivo*, respectively. The mitochondrial damage and oocyte apoptosis were measured, and the role of autophagy was validated in the *in vitro* experiment, with 3-MA, a commonly used inhibitor for autophagy.

The maturation of oocytes was found to be obviously affected by NRTIs in both the *in vivo* and the *in vitro* studies, as the oocyte maturation-related markers (MPF, GDF-9, TGF-β, IGF-1, Kit ligand, and PDK1) were all downregulated after the NRTI treatment. This is consistent with our further results, showing a reduced oocyte cleavage rate and blastocyst formation rate after the NRTI treatment. Further, the reduced mtDNA copy number, cAMP levels, and increased ROS levels were detected both *in vitro* and *in vivo*, indicating that mitochondrial toxicity was a major factor leading to the low fertility of mice treated with AZT and 3TC. All these alterations indicated an unstable intracellular environment and were critical components of cell apoptosis and autophagy [17–19, 24]. These inferences were confirmed by our further results, showing an increased oocyte apoptosis and upregulation of autophagy-related gene expressions such as ATG5, ATG7, and Beclin1, in both the *in vivo* and the *in vitro* studies.

To validate whether autophagy contributed to the low fertility of rat caused by AZT and 3TC, 3-MA, an autophagy inhibitor, was used in the *in vitro* study. Our data showed that 3-MA could partially attenuate the decrease in the mtDNA copy number and the ATP level, and the increase in oocyte apoptosis, after the AZT and 3TC treatment, suggesting that autophagy played a critical role in the NRTI-induced oocyte dysfunction and infertility. In contrast, the increase in the ROS level could not be attenuated; this may be attributed to the fact that ROS was the upstream activator of autophagy and cell death, and the production of ROS could not be suppressed by 3-MA [25, 26]. Levels of LC3-II are commonly used to monitor the autophagic process [27], and the mTOR level is negatively correlated to the autophagy progress [28]. In this study, we found that the AZT and 3TC treatments upregulated the LC3-II protein level, but the upregulation could be suppressed by 3-MA. Moreover, 3-MA could attenuate the downregulation of mTOR induced by AZT and 3TC, confirming that autophagy mediated the reduced fertility caused by NRTIs. Therefore, autophagy suppression may be a prospective treatment for the infertility of AIDS patients treated with NRTIs. Note that 3-MA did not attenuate but aggravate the NRTI-induced suppression of oocyte maturation, suggesting that autophagy could not account for the maturation disorder of oocytes caused by NRTIs. Therefore, NRTI-induced low fertility may be very complicated, and autophagy is only one of the related pathways.

In summary, our study showed that NRTIs decreased the fertility of female rats, leading to oocyte dysfunction including the suppression of oocyte maturation, decreases in the mtDNA copy number and ATP levels, and increases in the ROS levels and apoptosis. These alterations may be mediated by autophagy, whereas autophagy cannot account for the maturation disorder of oocytes caused by NRTIs. Therefore, we conclude that autophagy is one of the critical pathways in NRTI-induced female low fertility. Further research on the *in vivo* treatment effect of the autophagy inhibitor is required.

## MATERIALS AND METHODS

### Animals and treatment

12-week-old pathogen-free female Sprague-Dawley rats were obtained from Beijing Weitonglihua Biological Company. The rats were housed with a 12-h light/dark cycle under controlled temperature and humidity conditions; sufficient food and water were freely supplied. All the experiments were performed according to the international guidelines for animal research. This work was supervised and approved by the Institute of Animal Care and Committee of the first affiliated hospital of Kunming Medical University.

To investigate the effect of AZT and 3TC on rat fertility *in vivo*, the rats were randomly assigned to three groups: the control, AZT, and 3TC groups. The AZT and 3TC dosage used in this study was 12 mg/kg•day and 6 mg/kg•day for 3, 6, 9, and 12 weeks, respectively, with intragastric administration. Ten animals were used for each time point for each group. The dose was referenced from humans treated with HAART (600-mg AZT and/or 300-mg 3TC per day was used for AIDS patients weighing 50–70 kg). AZT and 3TC were bought from Daiichi Pharmaceutical Co., Ltd. (Beijing, China) and Anhui Biochem Co., Ltd. (Hefei, China), respectively.

To investigate whether NRTIs induced oocytes dysfunction was mediated by autophagy, 3-methyladenine (3-MA, Selleck Chemicals (Houston, USA)), a critical autophagy inhibitor was used for the *in vitro* study. The rats were assigned to the AZT, 3TC, AZT+3-MA, 3TC+3-MA, 3-MA, and control groups, respectively. The rats were intraperitoneally injected with 40 IU of pregnant mare serum gonadotropin (PMSG) (Folligon, Sigma–Aldrich, USA) to stimulate follicle development 46 h before sacrifice [13]. Oocytes were collected and treated with AZT, 3TC, AZT+3-MA, 3TC+3-MA, and 3-MA, respectively. 40-μM AZT and 30-μM 3TC were used to treat the rats oocytes *in vitro*; the doses were determined according to the corresponding content of the cord blood and the peripheral blood of pregnant women, as well as that of the peripheral blood of AIDS patients [29]. The concentration of 3-MA used in this study was 5 mM, as described in another study [30]. The oocytes were tested for maturation, mtDNA copy number, adenosine triphosphate (ATP) and ROS levels, apoptosis, and autophagy-related gene expressions in each group at 24, 48, and 72 h, respectively. Ten animals were used for each time point for each group.

### Oocyte collection

After the mice were killed, their ovaries were washed with phosphate-buffered saline (PBS) (Gibco, Applied Biosystems, USA). The droplet method was used to culture the rat oocytes under sterile conditions. The matured rat oocytes were rinsed with a balanced culture medium (Gibco, Applied Biosystems, USA) twice and then cultured in the 50-μL droplet with 4 g/L of bovine serum albumin (BSA) at 37°C, 5% O_2_, and 6.5% CO_2_ for 4 h; the medium was covered with mineral oil and balanced 6 h before use. The maturity of rat oocytes was estimated on the basis of the status of the cumulus–oocyte complexes.

### Oocyte maturation assays

To evaluate the maturation of the oocytes, the levels of maturation-promoting factor (MPF) [21], growth differentiation factor-9 (GDF-9) [20], transforming growth factor-β (TGF-β) [31], insulin-like growth factor-1 (IGF-1) [32], Kit ligand (KL) [33], phosphoinositide-dependent protein kinase 1 (PDK1) [34] were measured with commercial enzyme-linked immunosorbent assay (ELISA) kits provided by USCN business Co., Ltd (Wuhan, China), following the manufacturer’s instructions. Briefly, oocytes were homogenized in RIPA lysis buffer supplemented with protease inhibitors (Beyotime, Haimen, China) and centrifuged at 13,000 rpm for 15 min. The supernatants were collected for ELISA assay. The absorbance was measured on a microplate reader at a specific wavelength (Varioskan Flash, Thermo Electron, Finland), and the concentrations of MPF, GDF-9, TGF-β, IGF-1, Kit ligand, and PDK1 were calculated from a standard curve for each sample.

### *In vitro* fertilization

The mature oocytes were prepared for further fertilization. The sperm samples were obtained, prepared, and adjusted to the final concentration of 5 × 10^5^/mL for *in vitro* fertilization. 10 μl of the sperm suspension was admixed with oocytes and kept for 6 h according to a previously published study [35]. To remove the sperms from the surface of the oocytes, the oocytes were rinsed three times with the culture medium. Then, they were transferred to a G-IVF medium for overnight culturing at 37°C and 5% CO_2_. The fertilized oocytes were evaluated with double prokaryotic under a microscope. The intact surviving oocytes were evaluated by observing the homogeneous cytoplasm, transparent full rules, and membranes. The oocytes with unclear characteristics were omitted from further statistical analysis. The remaining GV-stage oocytes were deposited for further bench work. The related formulas were calculated as follows:

Cleavage rate=(No. of embryos/No. of surviving oocytes)×100%;

Blastocyst formation rate=(No. of blastocysts/No. of surviving oocytes)×100%.

### mtDNA copy number assay

A reduction of the mtDNA copy number is regarded as the most significant characteristic of HIV infection and the treatment of AIDS patients with NRTIs [36]. Thus, the mtDNA copy number of mice oocytes was measured with a real-time polymerase chain reaction (PCR) method in this study, by amplifying the conservative mitochondrial ND2 gene as described previously [36]. In brief, the total DNA was isolated with the Tiangen genome isolation kit (Tiangen Bitotech, Co. Ltd., Beijing, China), and the mtDNA gene fragments were amplified with the SYBR Premix Ex Taq TM II kit (TaKaRa, Dalian, China) according to manufacturer’s instructions [37]. The amplification of the β-actin gene was used for normalization.

### ATP activity and ROS detection

The cyclic adenosine monophosphate in oocytes was a derivative of adenosine triphosphate (ATP), which serves as the second important messenger in many biological processes. Further, cAMP signaling participates in the process of autophagy via a novel pathway including ERK, cyclin E, and Beclin 1 [38]. Thus, the level of cAMP was detected using luciferase. Further, the ATP level was measured with the ATP Assay Kit (Beyotime, Haimen, China) according to the manufacturer’s instructions. The ROS level in the mitochondria was detected with dichloro-dihydro-fluorescein diacetate (DCFH-DA) and probes (Beyotime, Haimen, China) as described in a previous work [39]. In brief, oocytes were washed three times with PBS buffer, then suspended with 10-μM DCFH-DA, and incubated at 37°C for 30 min. Finally, they were rinsed with the PBS buffer thrice. The fluorescence intensity was measured at the wavelengths of 488 and 525 nm.

### Quantitative real-time PCR

To evaluate the expression levels of genes related to autophagy and apoptosis, the total mRNA was isolated and reverse transcribed to cDNA (High Capacity cDNA Reverse Transcription Kit, Applied Biosystems™, USA), and the autophagy-related genes involving autophagy-related protein 5 (ATG 5), ATG 7, and Beclin 1 were evaluated as described in other studies [40, 41]. Apoptosis-related genes involving Bax and Bcl-2 were also examined according to our previous work [42]. Further, the mitochondria transcription factor (mtTFA) and nuclear respiratory factor 1 (NRF-1), were assayed to evaluate the mitochondrial functions. A real-time qPCR analysis was performed in triplicate using SYBR Green (Takara, Dalian, China) on a Light Cycler^®^ 96 System (Roche, Switzerland). These genes were all measured with real-time PCR by a comparison with the β-actin gene. The primer pairs used for real-time PCR are listed in Table 1.

**Table 1:** Primers used for PCR in this study

Gene	Sense	Anti-sense
ND2	ACTACCCGAAGTCACCCAAGGAAT	CAGGCGCCAACAAAGACTGATGAA
mtTFA	GAAAGCACAAATCAAGAGGAG	CTGCTTTCATCATGAGACAG
NRF-1	CTTTGGAGAATGTGGTGCGCAAGT	TCTGGGATAAATGCCCGAAGCTGA
ATG 5	ACTGCTTCGCTGAGACACACA	GCTTCGGCTGCATTGCAT
ATG 7	CGCCAAGATCTCCTACTCCAA	TTGCCACCCCCTAGACAATC
Bax	GCAGGGAGGATGGCTGGGGAGA	TCCAGACAAGCAGCCGCTCACG
Bcl-2	CGGGAGATCGTGATGAAGT	CCACCGAACTCAAAGAAGG
Beclin 1	GGCTGAGAGACTGGATCAGG	CTGCGTCTGGGCATAACG
β-actin	AGCCATGTACGTAGCCATCC	ACCCTCATAGATGGGCACAG

### Western blot analysis

To investigate whether autophagy mediated the oocyte dysfunction induced by AZT and 3TC, two critical proteins involved in autophagy, namely the microtubule-associated protein light chain 3 II (LC3-II) and the mammalian target of rapamycin (mTOR) [43, 44] were assayed using a Western blot analysis. In brief, the oocytes were homogenized in RIPA lysis buffer supplemented with protease inhibitors (Beyotime, Haimen, China). Then, the homogenates were centrifuged at 12000 rpm for 5 min, and the supernatant was the extracted proteins. The protein concentration was measured using the BCA kit (Beyotime, Haimen, China). Further, 40 µg of total protein of each sample per lane was separated by 12% SDS-polyacrylamide gel electrophoresis (PAGE) and transferred to the polyvinylidene difluoride membranes (Millipore, Germany). After blocking with 5% milk, the membranes were incubated with the primary antibodies: LC3-II (Abcam, ab48394, 1:1000 dilution), mTOR (Abcam, ab2732, 1:1000 dilution), and β-actin (Abcam, ab8226, 1:10000 dilution). After washing with TBST for 5 min × 3, the membranes were incubated with appropriate horseradish peroxidase-conjugated secondary antibodies (KPL, USA). Specific proteins were detected using an ECL kit (Beyotime, Haimen, China) and exposed to film. The protein expression level was analyzed with the AlphaEaseFC software (Alpha Innotech, USA), and the data were normalized using β-actin.

### Statistical analysis

Data were presented as mean ± sem. The relative mRNA, DNA, and protein levels in different groups were compared with one-way analysis of variance and least significant difference tests. The cleavage rate and the blastocyst formation rate among different groups were compared with a chi-square test. A two-sided *P*-value of < 0.05 was considered as statistically significant. The data analysis was performed using SPSS 17.0 (SPSS Inc., Chicago, IL, USA).

## Abbreviations and acronyms

NRTIs: Nucleoside reverse transcriptase inhibitors
AZT: zidovudine
3TC: lamivudine
AIDS: Acquired immunodeficiency syndrome
mtDNA: mitochondrial DNA
HAART: highly active antiretroviral therapy
ROS: reactive oxygen species
3-MA: 3-methyladenine
ATP: adenosine triphosphate
MPF: maturation-promoting factor
GDF-9: growth differentiation factor-9
TGF-β: transforming growth factor-β
IGF-1: insulin-like growth factor-1
PDK1: phosphoinositide-dependent protein kinase 1
ELISA: enzyme-linked immunosorbent assay
ATG5: autophagy-related protein 5
mtTFA: mitochondria transcription factor
NRF-1: nuclear respiratory factor 1
LC3-II: microtubule-associated protein light chain 3 II
mTOR: mammalian target of rapamycin
